# The Perpetual Pivot: Understanding Care Partner Experiences in Ontario Long-Term Care Homes during the COVID-19 Pandemic

**DOI:** 10.3390/geriatrics8050090

**Published:** 2023-09-08

**Authors:** Katherine Kortes-Miller, Maïa Natale, Kimberley Wilson, Arne Stinchcombe

**Affiliations:** 1 School of Social Work, Lakehead University, 955 Oliver Rd., Thunder Bay, ON P7B 5E1, Canada; 2 School of Psychology, University of Ottawa, Ottawa, ON K1N 6N5, Canada; 3 Department of Family Relations and Applied Nutrition, University of Guelph, Guelph, ON N1G 2W1, Canada

**Keywords:** caregiver, care partner, care recipient, long-term care home, social isolation

## Abstract

Long-term care homes (LTCHs) were impacted during the COVID-19 pandemic. With their ever-changing conditions and restrictions, care partners’ roles in LTCHs changed drastically. In this cross-sectional study, an electronic survey was used to examine the experiences of care part-ners who were caring for one or more adults in an Ontario LTCH during the pandemic. The survey was circulated through social media (convenience sample) which produced a convenience sample of 81 caregiver participants. Visit characteristics and a comparison in the quality of care in LTCHs was analyzed before the pandemic as well as during the most restrictive times. Visitation lengths and frequencies, other sources of communication such as phone and video calls, and various types of care provided by caregivers such as personal grooming and personal care all decreased significantly during the pandemic. Care partners also reported that the health of their care recipients decreased significantly during restrictive visitation times. Through thematic analysis, we identified three themes: restrictions and changing LTCH conditions created (1) social isolation and an erosion of connection, (2) a communication breakdown, and (3) a lack of person-centered care. Findings from this research can promote the health and wellbeing of residents and care partners within LTCHs.

**Key points:**

- Restrictions and changing long-term care home (LTCH) conditions created social isolation and an erosion of connection in LTCHs in Ontario during the pandemic
- Care partners reported that the health of their care recipients decreased significantly during restrictive visitation times
- Socialization needs to be valued when creating and enforcing public health protocols
- Open and regular communication between LTCHs and care partners is essential

## 1. Introduction

In December of 2019, the first cases of SARS-CoV-2 (COVID-19) were discovered and classified as a severe acute respiratory syndrome [1]. The virus that rapidly spread across the world was declared a pandemic by the World Health Organization (WHO) on the 11 March 2020. As is similar with any other contagious viruses, individuals who contract COVID-19 respond differently based on many factors including age and the pre-existence of health conditions [2]. For older adults, it is not uncommon to live with multiple chronic conditions, placing these individuals at greater risk of mortality and morbidity should they experience a COVID-19 infection [3]. Due to the congregate living and the additional exposure to healthcare staff and visitors, there was potential for long-term care home (LTCH) settings to enhance the risk to older adults who are already vulnerable to COVID-19 [4]. In Ontario, Canada, over 80% of deaths due to COVID-19 were within LTCHs [5]. The virus has, and continues to have, a significant impact on LTCHs with the occurrence of outbreaks. Research is needed to improve care, visitation policies, and the experiences of care partners and LTCH residents during outbreaks.

The initial spread and severity of the pandemic in 2020 and 2021 occurred over two waves, the first of which took place from 1 March to 31 August 2020 [6]. When case numbers rose once again, the second wave was declared between 1 September 2020 and 15 February 2021 [6]. During the pandemic, LTCH residents accounted for approximately 79% of COVID deaths in Canada during the first wave and 60% of deaths in the second wave [6]. Chu et al. [7] reported that government regulation and protocols were not prepared to address the rapidly growing needs of older adults in LTCHs, particularly around infection control at the onset of the pandemic. In an attempt to curb the spread of COVID-19 within LTCHs, administrations, following the guidelines issued by the government, enforced strict restrictions preventing family members and friends from entering the buildings and providing their usual care and visitation. Chu and colleagues’ [7] research highlighted that very little information was shared with families and caregivers about the timelines of these restrictive visitation policies, resulting in increased social isolation and emotional harm to residents and caregivers.

After months of advocacy from across sectors, including family and friends of residents living in LTCHs, visitation restrictions within LTCHs slowly shifted, partially in response to public scrutiny. As Palubiski et al. [8] acknowledge, with the pandemic being a novel and unprecedented situation it was necessary to reintegrate family, friends, and essential caregivers back into LTCHs in a timely manner. By summer 2020, outdoor visits were facilitated and by the fall of 2020 family members were allowed to enter LTCHs and to visit residents indoors. In September 2020, the Ontario government created a designation of “essential caregiver” to up to two individuals per LTCH resident. Before this, as Chu et al. [7] reported, family caregivers were often viewed as visitors, and were therefore not “essential”. The designation of “essential caregiver” would retain visitation rights regardless of broader COVID-19 restrictions. This shift in policy also indicated a recognition of the integral role of caregivers in resident well-being, including representing the care recipient’s desires and history, as well as maintaining social connections between residents and their families/friends [9]. Essential caregivers often play the role of substitute decision maker and power of attorney, designations that allow people to make decisions for a person who is incapable of making medical decisions for themselves [10].

Within our study we use the term care partner as defined by Healthcare Excellence Canada [11]. Care partners may be a family member, friend, or neighbour. Palibiski et al. [8] extend this idea to “Essential Caregivers” who provide personal, social, psychological, and/or physical support and assistance as recognized by the resident to be essential to their well-being in LTC [8]. Badone [12] also notes that responsibilities for many of the care duties, including feeding and physical activity in LTCHs, had fallen to the residents’ visiting friends and family because of low staffing and resources, and this state only worsened during the pandemic which served to increase both the partnership in caregiving and the essential nature of the care.

The purpose of our study was to examine the experiences of care partners in Ontario to inform future research on the best practices to promote the health and wellbeing of this population during periods of outbreak similar to the COVID-19 pandemic. Care partner experiences, significant differences in visit characteristics before and during the pandemic, and the quality of care during restrictions were also explored. Using data collected via an electronic survey, the following two research questions were examined: (1) How were adults living in long term care facilities in Ontario affected by the COVID-19 pandemic? And, (2) How were caregivers of adults living in LTCH in Ontario affected by the COVID-19 pandemic?

## 2. Method

We analyzed data from a cross-sectional survey designed to explore care partners’ experiences during the COVID-19 pandemic. A convenience sample of 81 caregivers participated in the study; participants were primarily recruited using social media between April and August 2021. The eligibility criteria encompassed individuals over the age of 18 who served as care partners for individuals residing in a LTCH in Ontario, Canada, at any point from March 2020 to March 2021.

The survey captured participant and care recipients’ demographic information (see Table 1), characteristics of the care recipients, a comparison of care before and during the pandemic, as well as care partner experiences. The goal of the survey was to gain an insight in the experience of care partners of those living in a LTCH in Ontario during the COVID-19 pandemic in order to explore the questions, concerns, and expectations around LTCHs as well as inform the ways that care partners were maintaining connection during social isolation.

Participants gave informed consent prior to completing the survey. This survey received approval from the Research Ethics Board from [redacted] University (following the Canadian Tri-Council Policy Statement: Ethical Conduct for Research Involving Humans) and was conducted on the Qualtrics platform. A “prefer not to answer” response option was offered in cases where participants did not feel comfortable responding. As compensation for involvement in the study, participants were entered into a draw to win one of four $50 grocery gift cards.

The survey was divided into three sections. The first section collected demographic information and participants were asked to provide information such as their gender, age, and details regarding their care recipient. For example, with respect to information about the care recipient, participants were asked “How many individuals are you caring for?”.

The second section asked caregivers to compare their experiences as well as the quality of care in the LTCH before the pandemic versus during the most restrictive visiting period of the pandemic. Each question had two answers to be filled out, one for “before the pandemic”, and the other for “during the most restrictive time”, which was defined as the general period during the pandemic when restrictions were at a high for each care partner’s geographical area and LTCH. The questions included differences in the frequency of visits before and during the pandemic, the frequency of LTCH communication with care partners, and the quality of activities and care offered to care recipients. For example, questions included: “Did your care recipient have additional people (such as a paid companion, podiatrist, physical therapist, or hairdresser) provide care or services to them either in or out of the care home?” with response options of “Yes”, “No”, “I do not know/I am unsure”, and “Prefer not to answer”.

Finally, participants were asked open-ended questions where they described their needs as a care partner, what they wished was different about the care during the pandemic, their overall experience as care partners during the pandemic, and any personal stories that were not otherwise told through the rest of the survey. For example, participants were asked “What do you want long-term care decision-makers to understand most about your experience as a caregiver during the pandemic”?

## 3. Analysis

The analysis of these data is separated into the following: (1) care partner and care recipient demographic characteristics; (2) the comparison of visit characteristics before the pandemic versus during the most restrictive times; and (3) the comparison of quality of care before the pandemic versus during the most restrictive times. Descriptive statistics, including means, standard deviations (SD), and frequency were computed. McNemar’s test was used to examine the change from before the pandemic to the most restrictive time.

The qualitative responses from the four open-ended questions were analyzed following Braun and Clarke’s approach to an inductive thematic analysis [13]. This analysis took a constructivist, inductive experiential semantic approach. Braun and Clarke’s [14] six-step analytical process was then used to better familiarize the research team with the data and obtain an overall understanding of responses. The responses were then grouped into common codes, identifying common trends, or central organizing concepts, and eventually reconfigured through numerous iterations of codes to generate concise themes. These themes allowed the researchers to concisely illustrate the experiences of Ontario-based caregivers and ECPs. M.N and A.S led the analysis with input from the other research team members.

## 4. Findings

### 4.1. Quantitative Findings

Participants’ demographic characteristics are presented in Table 1. The majority of caregivers who participated were women (90.8%), with an average age of 59.9 years old (range: 18–78 years). The care partner’s relationship to the survey participant varied from spouse to parent, child, grandparent, family/relative, client, or friend, with the majority of participants identifying as the child of their care recipient (62%). Just over one quarter (28.4%) of survey participants were named as the Substitute Decision Maker for their care recipient. The majority of the survey participants (62%) indicated they were designated as Power of Attorney. Their yearly household income was spread amongst less than $50,000 CAD to more than $100,000, with over 83% of the sample reporting a household income of less than $100,000. Education levels amongst those who completed the survey ranged from a high school diploma to completion of a graduate degree, with the majority (67.4%) reporting achieving a college or university degree. Finally, the average distance from the survey participant to their care recipient was 29.7 km.

The second section of Table 1 provides insight on the demographic characteristics of the care recipients. Most (67%) recipients were women and had an average age of 85. The LTCHs were spread across Ontario, but the large majority (80%) were located in Northern and Southern Ontario. When asked about which period of the pandemic was most challenging for care recipients, they mostly (49.3%) stated that no time appeared to be more difficult than another, and rarely (3.7%) claimed that the onset of the pandemic (March 2020) was the most challenging.

The characteristics of care partner visits before and during the pandemic were compared using McNemar’s test for significance. The results showed that there was a significant difference in visitation frequency, with the majority being several times per week or more before the pandemic and every other week or less during the most restrictive times (X^2^ = 35.4, *p* ≤ 0.001). The results show that visit durations decreased significantly (X^2^ = 21.4, *p* ≤ 0.001). The frequency of various types of care provided by care partners, such as personal care (*p* ≤ 0.001), personal grooming (*p* ≤ 0.001), and laundry (*p* ≤ 0.006), etc., decreased significantly. There was also a significant increase in video communication (X^2^ = 28.5, *p* ≤ 0.001) during the most restrictive times. These results are summarized in Table 2.

As demonstrated in Table 3, there was a significant decrease in the care partner’s assessment of the recipient’s health (X^2^ = 45, *p* ≤ 0.001), as well as in the quality of care at the LTCH (X^2^ = 32, *p* ≤ 0.001). Care partners reported feeling significantly less up to date about their recipient (X^2^ = 4.4, *p* ≤ 0.001), which is in accordance with the overall reported decrease in the frequency of communication from the LTCH (X^2^ = 7.7, *p* = 0.264). Lastly, care partners felt significantly less involved in both the care and decision making of their care recipient (X^2^ = 26.8, *p* ≤ 0.001).

### 4.2. Qualitative Findings

Through thematic analysis, three themes were identified: social isolation and an erosion of connection, a communication breakdown, and a lack of person-centered care. These themes capture the experiences of the care partners and their perception of care recipients during the most restrictive times of the pandemic.

#### 4.2.1. Social Isolation and an Erosion of Connection

Participants described the immense social isolation faced by both the care partners and care recipients in Ontario LTCHs. The increased restrictions resulted in a major reduction in visitations, as well as the perception of decreased quantity and quality of care lessened care by LTCH staff. With the restrictions on in-person visits came the necessity for care recipients to use technology to connect with their care partner. However, many participants reported that often their care recipient was unable to use the technology because of memory issues associated with dementia or Alzheimer’s, as well as a lack of support with technology within the LTCH. One participant experienced prolonged delays on the phone when the resident attempted to seek assistance from staff, which was ultimately unsuccessful:

*[…] My mom told me she felt isolated and forgotten. I think decision-makers need to remember that many older adults aren’t tech-savvy and due to staffing shortages, it was not always possible to Facetime with my mom or even have a phone call.*

As a result, many experienced prolonged periods of isolation due to their inability to use the technology and the lack of in-person support from care partners. This created yet another barrier to communication and connection with people outside of the LTCH.

A common description of the conditions of visitations created an environment that many participants described as being similar to a prison:

*Then when she gets agitated because she doesn’t want to stay in her room [they] put a gate up. She tried climbing over that as she didn’t “understand she was a prisoner”… then … to keep her confined to her room, [they] put her in a wheelchair with her feet off the floor … to sit out COVID.*

Participants reported that residents felt neglected, isolated, confused, and angry with how the LTCH was implementing protocols and the lack of help offered. Survey participants also frequently reported observing rapid health declines in their care recipients following the lack of visits and socialization. This demonstrates that social connection is essential to the mental and physical wellbeing of the aging population.

One example of isolation within the LTCH occurred between three siblings. While they were living in the same LTCH, they were separated for months without explanation leaving them feeling helpless in their efforts to be reunited. Efforts by their care partner were also not successful:

*The infection control policies/procedures have kept my 3 care recipients from visiting with each other since they individually reside in different wings of the same long-term care home—although they are siblings, they are not allowed to visit each other since pandemic started.*

The survey participants frequently described the residents’ living situations, in which they were isolated from the rest of the residents, and very rarely had the opportunity to socialize, go outside, etc.:

*I was allowed as a pilot test to visit with my mom outside and she asked me if she was allowed out in the area. I asked ‘why mom’, and she said, ‘because I am always in the dark’! This is when I became more vocal about the need to get beyond her four walls, to increase her view, give her fresh air, provide us with more stimulus for communication, improve her outlook and quality of remaining life.*

Several participants stated that they observed severe decreases in their care recipient’s health due to their lack of socialization. One participant explains the direct link between social isolation and a health decline:

*He needs more personal contact. He is lonely and it is making him sick. His loneliness is literally killing him. He needs recreation staff or pastoral care to spend a few minutes with him.*

#### 4.2.2. Communication Breakdown

Through our analysis, it was clear that care partners faced challenges in reaching LTCHs, which made it challenging and frustrating for them to receive essential information on the care and the state of their care recipient(s). Survey participants reported seldom obtaining the necessary updates to alleviate their concerns. This, paired with the inability to reach residents over the phone, over a video call, or at an in-person visit, created an environment of uncertainty and worry for participants:

*As a family member you can’t see the care that is being provided you have to trust that it is being done. You don’t really know what is going on. You don’t see if meals have changed, activities have changed, etc.*

Although participant responses suggested there were similar amounts of communication from LTCHs before and during the pandemic, care partners felt much less aware of the health of the resident during restrictions; 52.6% always felt up to date about the care recipient before the pandemic and 36.7% felt up to date during the most restrictive times. There was also a significant drop in the care partner’s ability to provide suggestions or requestions regarding the care of the recipient. Most communication from the LTCH was more general in nature and answered less of the participants’ questions than before COVID. It can be said then that the perceived quality of communication between the LTCH and survey participants dropped significantly.

Care partners wanted more involvement in decision making processes than LTCHs allowed. Survey participants suggest that their unique perspective of the care of their recipient, based on their personal history, makes their approach one that should be considered when determining important changes for residents: *“Family members know better how to care for the people we love, & they [LTCHs] should listen & respect what we explain to them”.* Supporting the perspective that care partners should be more involved in the decision making in LTCHs, another participant suggested that:

*Family members ought to have a greater role in decision making regarding their loved ones. There needs to be a balance between guarding against the potential harm of contracting physical illness versus the actual harm produced by extreme isolation (from family members, volunteers and from other residents).*

Survey participants expressed a desire for more forward communication that would make it easier to receive and understand information about their care recipient. As one participant states, they need *“… more communication with the management of the home. More staffing as it is a HUGE issue and getting answers is almost impossible”.* The fear that arises when care partners cannot reach the resident or receive an update on their state is extremely impactful. The lack of communication can create high levels of stress for both the care partner and the care recipient:

*Family members need to know their loved one is okay mentally as well as physically. Without getting inside the home, we have no idea about the quality of care being given or the actual wellbeing of our loved one. “He’s doing fine” is not enough.*

#### 4.2.3. Lack of Person-Centered Care

The participants also reported a lack of person-centered, compassionate, and adequate care for residents. They noted how the reduced and insufficient staffing complement often fell short in terms of maintaining residents’ personal hygiene and overall inability to see to every individual resident’s needs. Since opportunities for visits were extremely limited in the most restrictive times, survey participants reported relying on formal care workers to support their care recipients. Confidence was lost when they eventually heard reports from the residents detailing the conditions of care that they endured during their daily activities. This left survey participants extremely concerned and feeling powerless to do anything to decrease these worries. Once again, they reported feeling helpless and as though the LTCH did not have an inclusive approach to care that valued their contributions and involvement in the well-being of their care recipient. Many participants shared their shock in the neglect that residents faced:

*I seem to be the only person that can care for him. The nursing staff leave his care for me to do and he stays all day and night in the clothes I put him in. Not changed, teeth brushed, no showers.*

The participants supported the notion that an increased staff would lead to more attentive care. When asked what they would hope for the future of care in LTCHs, one participant stated that they wish there was:

*[…] enough staff for her to be treated as an individual, rather than a box on a list of tasks that needs to be checked off. I want staff to be paid more, and to have the necessary training, so that they can better recognize the individual needs of all the patients, and tailor their interactions accordingly. It is beyond ridiculous that patients are being lined up in the halls sometimes upwards of an hour before mealtimes begin so that they can be all taken in together.*

The participants also shared that the mental health of their care recipient was not considered when creating restrictions or carrying out daily activities with the residents. One participant stated that they would like LTCHs to know that:

*There are still family caregivers that really love their loved ones and are very concerned for their health and mental health … and that staff treat them with respect […]*

Throughout the survey, the participants urged LTCHs to consider adopting a more compassionate and attentive approach to care to increase the quality of life that the residents have left to live and reduce the worry faced by care partners.

## 5. Discussion

Care partners have a crucial role in supporting residents in LTCHs in Ontario and this was no more evident than during the pandemic. The findings from this study will resonate with people who became more aware of the challenges facing LTCHs and the people accessing their care brought to the forefront during COVID-19. Specifically, this study examined the experiences and concerns of care partners of residents in LTCHs in Ontario, Canada, during the COVID-19 pandemic. The survey participants reported that their care recipients felt elevated levels of social isolation and loneliness during restrictive visitation times in the LTCH throughout the pandemic. This finding aligns with the research conducted by Beogo et al. [15], which suggested that restrictions during the pandemic exacerbated feelings of isolation among LTCH residents. Similarly, the findings from our study also align with the work of Smaling et al. [16] who found that the effects of COVID distancing measures on caregivers and health professionals resulted in a deterioration of the care recipient’s health, alongside an emergence of many new struggles faced by caregivers. These new struggles included difficulties adapting to the visiting restrictions and anxiety emerging from concerns about their recipient’s health and changes in the levels of care. Our study found similar indicators that suggest that restrictions led to feelings of worry, as many participants expressed a distress while caring for a resident in a LTCH during the pandemic. Qualitative accounts highlighted the communication breakdown with survey participants experiencing additional stress related to poor communication from the LTCH and sufficient updates about their care recipient. These consistencies within the existing literature suggesting that caregivers experienced more distress related to the care of their care recipient during the pandemic, resulting in a worsened experience for both the survey participants and their care recipients in LTCHs during times of COVID restrictions.

Badone [12] noted that responsibility for many of the care duties in LTCHs had fallen to family members and other caregivers because of low staffing and resources, and that low staffing in LTCHs has only worsened during the pandemic. This underscores the importance of care partners as they are often very involved in the care process of residents and suggests that care partners may offer an intimate and valuable perspective to the residents’ experiences and needs. The significant changes in the quality of care during the pandemic, as outlined in Table 3, point towards the need for a different approach to public health restrictions in LTCHs.

Bethell et al. [9] found that loneliness and isolation increased across the aging population throughout the COVID-19 pandemic. Descriptions of loneliness faced by both survey participants and care recipients during times of increased restrictions align with Bethell et al.’s [9] research. Bethell et al. [9] also reported that socialization decreased during the pandemic restrictions, whilst highlighting that socialization was an area of concern in LTCHs before the pandemic. The restrictions enforced by the provincial government and LTCHs further increased the number of older adults experiencing feelings of isolation and loneliness [15]. Munshi et al. [17] reported that restrictive visitor policies were associated with potential harms across specific populations including residents in LTCHs. Our study further encourages LTCHs and decision makers to value socialization when creating and enforcing public health protocols, as we have demonstrated a potential link between restrictions that limit human connection and a breakdown in communication and relationships, as well as a negative impact on the experiences of caregivers and care recipients in LTCHs.

The findings from our study also align with research from Norway by Smaling et al. [16] which highlighted the negative impact the pandemic had on family caregivers with the ever-changing restrictions and lack of communication and visitation. Smaling et al. [16] reported that as the challenges of the pandemic continued, communication issues with family members increased, leaving people confused, anxious, and at a loss of what to do next as the pandemic continued.

Our research provides an in-depth look at the experiences of care partners of residents in Ontario LTCHs, but as views and opinions vary by person and by city or province, it cannot be assumed that these results will be congruent with those that would be observed in other Canadian provinces or territories. The results of this research rely on the participants’ honesty and ability to be introspective to produce accurate submissions during a very challenging time. It is evident through the analysis of our data that participants tended to have negative experiences with LTCHs throughout the pandemic which was also confirmed before the pandemic [12] and in different provinces including British Columbia [18].

There have been longstanding critiques regarding quality of care within LTCHs across Canada that precede the pandemic [12,18]; it is evident through the analysis of our data that the pandemic served to exacerbate negative experiences with LTCHs for participants.

Building on our findings, in future research it would be beneficial to study the LTCH quality of care and care characteristics at a third time interval: post visitor restrictions. By measuring care partners’ experiences after a significant amount of time had passed since the pandemic and restrictions had begun to ease, we could better inform the specific variations observed and measure the true impact of the pandemic restrictions. If caregivers reported that these factors were found to return to pre-pandemic conditions once restrictions had been lifted or eased (i.e., a return to an improved quality of care and increased frequency/duration of visitations in the home), this could further conclusively support the finding that the COVID-19 restrictions were the primary factor in worsening conditions in LTCHs.

**Recommendations to improve caregiver support in LTCHs:** As Ontario moves into a period where COVID-19 is less prevalent, it is a time for reflection and action to develop strategies that will serve to decrease the negative experiences encountered by both care providers and care recipients in LTCHs during the height of the pandemic. The findings from this study inform the following three recommendations.

**Prioritizing Communication with Care Partners**: Consistent and transparent communication between care providers and LTCHs is essential during a pandemic or future lockdowns for sharing critical information and maintaining connections between residents and their care providers. Communication protocols should be put into place before a lockdown so care providers know what to expect and can have virtual communication tools in place if required. It is important that this communication be both compassionate and flexible.

**Embedding the essential role of care providers into institutional care practices**: LTCHs should acknowledge the critical care provided for residents by their families and friends as integral to care plans. Care providers should have access to LTCH residents because of the foundational role they play even during a high rate of transmission during a pandemic or outbreak [8]. Increased collaboration and consultation between the care providers and LTCHs may assist in improving the care provision experiences for all parties during times outside the pandemic.

**Recognize and Prevent Social Isolation:** Increased social isolation was an unintended adverse consequence associated with public health measures during the pandemic; concurrently, social isolation is a known risk factor for a myriad of deleterious health outcomes for older people. LTCHs in partnership with care providers should develop and promote innovative solutions to combat social isolation during periods of heightened public health restrictions (e.g., influenza), including virtual visits, online activities, and safe outdoor visiting options. Care providers need to be involved in the creation of these solutions that best fit the needs of their care recipient.

## 6. Conclusions

Care partner contributions are fundamental to supporting residents in LTCHs [19]. Our findings highlight some of negative effects that the COVID-19 restrictions in LTCHs had on care partners and care recipients including a decrease in visitations and communication, reports of worsening health, poor experience with staff at the LTCH during restrictions, and the overall decrease in the quality of care of the home. The need for improved care and communication in LTCHs is evident. As Beogo et al. [15] noted, social isolation was already an issue for the aging population before the pandemic and became what should be a call to action for LTCHs to address this. Given these effects, future research is required to inform best practices to further promote the health and wellbeing of caregivers, care recipients, and LTCH workers. Residents in long-term care homes will always be vulnerable to infectious pathogens. This research has shown that moving forward it is integral for LTCHs to work in partnership with care partners to ensure essential care is provided during times of outbreak and beyond.

## Figures and Tables

**Table 1:** Caregiver and care recipient demographic characteristics.

	Variable	Mean (SD) or % (n)
Caregiver		
	Gender	
	Woman	90.8% (70)
	Man	9.2% (7)
	Age	59.9 (11.6)
	Recipient’s relationship to caregiver	
	Spouse	10.1% (8)
	Parent	62% (49)
	Child	5.1% (4)
	Grandparent, family/relative, client, or friend	15.2% (12)
	Other	7.6% (6)
	Designated substitute decision maker	28.4% (23)
	Designated power of attorney	62% (50)
	Yearly household income	
	<50,000	33.9% (22)
	50–75,000	24.7% (16)
	75–100,000	24.7% (16)
	>100,000	16.7% (11)
	Education	
	High school diploma or less	20.9% (16)
	College or university diploma	67.4% (52)
	Graduate degree	11.7% (9)
	Distance to care recipient (km)	29.65 (61.5)
Care recipient		
	Gender	
	Woman	67% (51)
	Man	33% (25)
	Age	84.9 (9.3)
	Geographical area	
	North-Eastern Ontario	17.5% (14)
	North-Western Ontario	27.5% (21)
	Central Ontario	15% (12)
	Southern Ontario	35% (27)
	Other	5% (4)
	Most challenging period for recipients	
	Onset of the pandemic (March 2020)	3.7% (4)
	The first few months (April/May 2020)	27.2% (23)
	Summer 2020 and onwards	19.8% (13)
	Other/no time appears to be more difficult than another	49.3% (38)

**Table 2:** Comparison of visit characteristics before the pandemic versus during the most restrictive times.

	Response Options	Before the Pandemic %	During the Most Restrictive Times %	X^2^ (*p*)
Visitation Frequency				35.4 <0.001
	Daily	22.4	9	
	Several times per week	55.3	16.4	
	Weekly	15.8	26.9	
	Every other week or less	6.5	47.7	
Visitation duration				21.4 (<0.001)
	Less than one hour	18.7	43.1	
	One to two hours	33.3	5.9	
	Two to four hours	40	7.8	
	More than four hours	8	43.1	
Types of care provided by caregiver				
	Personal care (bathing, dressing, help to the toilet)	30.9	12.3	0.001
	Personal grooming (shaving, nail care, hair combing)	60.5	16	<0.001
	Laundry	16	3.7	0.006
	Medical care	13.6	3.7	0.008
	Encouragement to eat/drink	58	25.9	<0.001
	Walking or exercise	60.5	7.4	<0.001
	Reading or educating on current events	59.3	9.9	<0.001
	Taking care recipient for social outings	55.6	4.9	<0.001
Frequency of phone calls				6.6 (0.247)
	Several times per week	43.1	35.3	
	Weekly	5.9	5.9	
	Less than weekly	7.8	5.9	
	Not at all	43.1	52.9	
Frequency of video-calling				28.5 (<0.001)
	Several times per week or more	8.7	15.3	
	Weekly	11.6	34.7	
	Every other week or less	5.8	18.1	
	Not at all	73.9	31.9	

**Table 3:** Comparison of quality of care before the pandemic versus during the most restrictive times.

	Response Options	Before the Pandemic %	During the Most Restrictive Times %	X^2^ (*p*)
Caregiver’s assessment of recipient’s health				45.0 (<0.001)
	Very poor	2.7	32	
	Poor	14.7	25.3	
	Fair	28	24	
	Good to Excellent	54.6	18.7	
Quality of care at the LTCH				32.0 (<0.001)
	Poor to very poor	6.7	31.4	
	Fair	24	28.6	
	Good	26.7	15.7	
	Very good to excellent	42.7	24.3	
Caregiver felt up to date about the care recipient				24.4 (<0.001)
	Always	52.6	36.7	
	Sometimes	36.8	31.6	
	Rarely or less	10.6	31.7	
Frequency of communication from the LTCH				7.7 (0.264)
	Every day	16.7	12.7	
	Once per week or every other week	38.9	30.9	
	Once a month to once every few months	22.2	21.8	
	No communication	22.2	34.5	
Desired involvement in the care of recipient				40.0 (<0.001)
	Always	64	21.1	
	Sometimes or rarely	32	46.1	
	Never	4	32.8	
Desired involvement in decision-making				26.8 (<0.001)
	Always	62.7	35.1	
	Sometimes or rarely	26.7	21.6	
	Never	10.6	43.3	

## Data Availability

The data are not publicly available due to Research Ethics Board (REB) protocol.
